# Nerves and availability of mesodermal cells are essential for the function of the segment addition zone (SAZ) during segment regeneration in polychaete annelids

**DOI:** 10.1007/s00427-024-00713-5

**Published:** 2024-02-10

**Authors:** Benoni Boilly, Hubert Hondermarck, M. Teresa Aguado

**Affiliations:** 1 Département de Biologie, Université de Lille, 59650, Villeneuve d’Ascq, France; 2 Hunter Medical Research Institute, School of Biomedical Sciences & Pharmacy, College of Medicine and Wellbeing, University of Newcastle, Callaghan, New South Wales 2308 Australia; 3 Biodiversitätsmuseum, Georg August University, Untere Karspüle, 2, 37073 Göttingen, Germany

**Keywords:** Regeneration, Segment regeneration, Nerve dependence, Regeneration cell, Cell proliferation, Syllidae, Nereidae, Aricidae

## Abstract

Most of annelids grow all over their asexual life through the continuous addition of segments from a special zone called “segment addition zone” (SAZ) adjacent to the posterior extremity called pygidium. Amputation of posterior segments leads to regeneration (posterior regeneration-PR) of the pygidium and a new SAZ, as well as new segments issued from this new SAZ. Amputation of anterior segments leads some species to regeneration (anterior regeneration-AR) of the prostomium and a SAZ which produces new segments postero-anteriorly as during PR. During the 1960s and 1970s decades, experimental methods on different species (Syllidae, Nereidae, Aricidae) showed that the function of SAZ depends on the presence and number of mesodermal regeneration cells. Selective destruction of mesodermal regeneration cells in AR had no effect on the regeneration of the prostomium, but as for PR, it inhibited segment regeneration. Thus, worms deprived of mesodermal regeneration cells are always able to regenerate the pygidium or the prostomium, but they are unable to regenerate segments, a result which indicates that the SAZ functions only if these regeneration cells are present during PR or AR. Additionally, during AR, nerve fibres regenerate from the cut nerve cord toward the newformed brain, a situation which deprives the SAZ of local regenerating nerve fibres and their secreted growth factors. In contrast, during PR, nerve fibres regenerate both during the entire regeneration phase and then in normal growth. This review summarizes the experimental evidence for mesoderm cell involvement in segment regeneration, and the differential impact of the digestive tube and the regenerated nerve cord during PR vs AR.

## Introduction

**Fig. 1:**
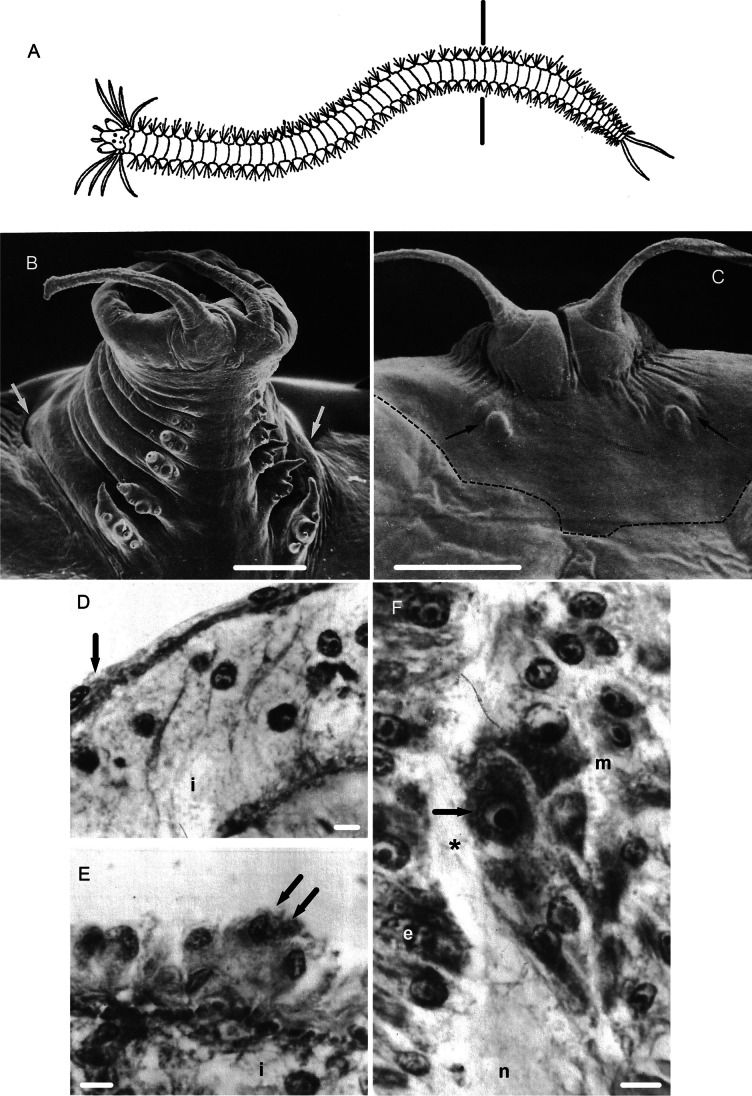
**A** Schematic drawing representing a Polychaete annelid **(***Nereis* sp. dorsal view) showing, antero-posteriorly, the prostomium (with its 2 antennae, 2 palps and 4 eyes), the peristomium (with its 4 pairs of tentacular cirri), and the following setigerous segments (with each a pair of parapodia characterized by a pair of cirri and bristles), which extends until the last part of the worm corresponding to pygidium (with its 2 anal cirri). **B** Posterior regenerate (ventrolateral view) of *Hediste diversicolor* (formerly *Nereis diversicolor*) 25 dpa (SEM picture) composed of the pygidium with its 2 anal cirri and 7 differentiating setigerous segments (indicated by arrows); the last one, close to the pygidium, shows only the first stage of differentiation as a bulge corresponding to the future parapodium. The space between this last differentiating segment and the pygidium corresponds to the SAZ. The SAZ is localized between the pygidium and the first sign of metamerization. **C** Posterior regenerate (ventral view) of decerebrated *Hediste diversicolor* 40 dpa; only the pygidium is completely regenerated but the presence of a pair of bulges (arrows) corresponding to the first phase of segmentation indicates that this regeneration phase fails. The dotted line indicates the limit of the regenerated area. **D**, **E** Mesodermal regeneration cells (*Syllis amica)*. Partial view of the coelomic epithelium surrounding the intestine in the amputated segment (2 dpa) showing their swelling (double arrow in **E**) compared to their flat aspect in non-injured segments (single arrow in **D**) i: intestine. **F** Postero-ventral extremity of the blastema 3 dpa (PR) close to the regenerated nerve cord (n) between the mesodermal regeneration cell mass (m) and the regenerated epidermis (e); activated mesodermal cells (arrow) close to the regenerated nerves (asterisk). **A** modified from Boilly et al. (1975); **B** and **C** modified from Boilly (1974); **D** and **E** modified from Boilly (1967a). Scale bars 200 μm (**B**, **C**), 5 μm (**D**–**F**)

**Fig. 2:**
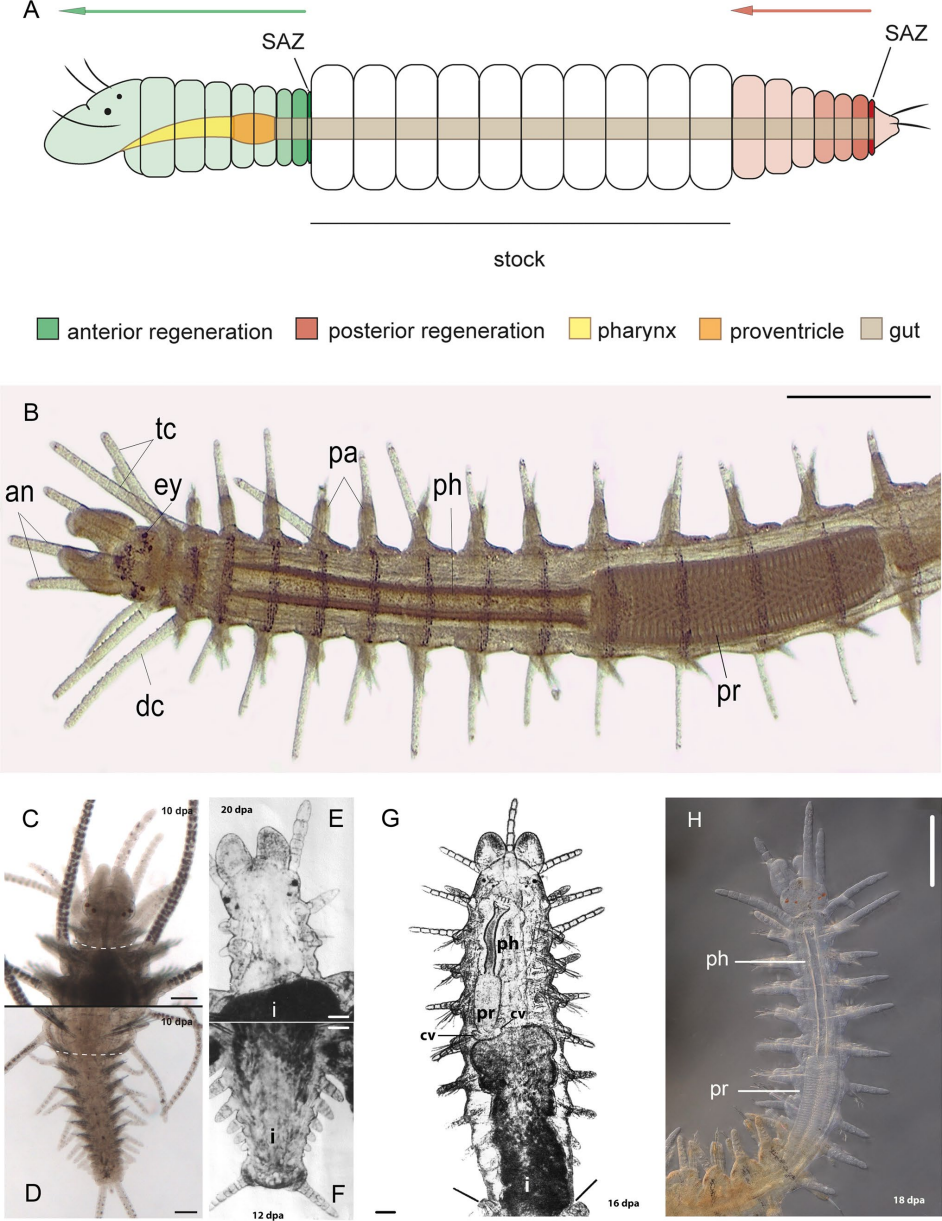
**A** Schematic drawing representing anterior and posterior regeneration of syllids from a midbody cutting area; SAZ, segment addition zone; arrows indicating the direction of regeneration from each SAZ. **B** Anterior end of *Syllis malaquini* showing by transparency the complex anatomy of the anterior digestive tube of stomodeal origin (a specificity of *Syllis*) composed of 3 parts: pharynx (ph), proventricle (pr), ventricle (vc). The head (an, antenna; ey, eye; pl, palp; tc, tentacular cirri), first segments (dc, dorsal cirrus; pa, parapodium) and posterior body part (not shown in this picture) are similar to *Nereis*. **C** Anterior regenerate of *Typosyllis antoni* (10 dpa), limited to prostomium and peristomium (no setigerous segments regenerated) and incomplete intestine (proventricle and pharynx missing). **D** Normal posterior regenerate of *Typosyllis antoni* (10 dpa). **E** Anterior regenerate of *Syllis amica* (20 dpa), prostomium, peristomium regenerated as well 2 setigerous segments but incomplete intestine (proventricle and pharynx missing). The intestine (i, black) did not regenerate and did not penetrate into the regenerate. **F** Normal posterior regenerate of *Syllis amica* (12 dpa), the intestine (i) is regenerated towards the pygidium. **G** Anterior regenerate of *Syllis gracilis* (dorsal view) (16 dpa) composed of prostomium, peristomium and 8 setigerous regenerated segments. Note that the intestine (i) present inside the regenerate is extended anteriorly by the regenerated anterior digestive tube (of stomodeal origin) composed of (not black): pharynx (ph), proventricle (pr) and ventricle with caeca (vc). **H** Anterior regenerate of *Syllis malaquini* (18 dpa) composed of prostomium, peristomium and 7 setigerous segments with pharynx (ph) and proventricle (pr). **A** modified from Ribeiro et al. (2018); **B** from Ribeiro et al. (2020); **C** and **D** from Weidhase et al. (2017); **E** and **F** from Boilly (1967a); **G** from Boilly and Thibaut (1974); **H** picture taken by Vanessa Spieß*.* Scale bars 1 mm (**B**), 100 μm (**C**, **D**), 50 μm (**E**–**G**), 200 μm (**H**)

Growth control is essential for life and relies on the presence of cells able to proliferate under growth factor stimulation. This applies to embryogenesis and post-natal development, regeneration and also pathological situations such as cancer. Because of the potential applications in regenerative medicine and cancer therapy, research on post-embryonic growth and especially regeneration is of great interest. Regeneration represents a good study model of growth (Abeloos 1932) and is an amazing capacity of some animals, especially many invertebrates able to reproduce embryonic processes after amputation in the adult. Among invertebrates, marine annelids, especially Errantia, which are well known for their high regeneration capacity, offer numerous research possibilities. In contrast to many regenerating animals, most polychaete annelids grow continuously (sexualization period excepted) during life because of a continuous production of new segments from a special area adjacent to the posterior-most part of the worm (the pygidium), called the segment addition zone (SAZ) (Balavoine 2014). These animals represent a good regeneration research model because of the relative simplicity of their anatomy i.e. a succession of similar segments between the posterior pygidium and anterior prostomium (Figs. 1A, 2A). Moreover, in some species, regeneration of the same body parts is controlled differently according to various parameters like body polarity for instance (Boilly et al. 2017a, 2017b, 2022).

New segments produced from the SAZ will differentiate postero-anteriorly in such a way that the worm lengthens during its non-reproductive life stages (Fig. 1A). This phenomenon may find a correspondence with the developmental elongation process of the trochophore (Nielsen 2005). The trochophore is a motile larval form of embryonic development with a spinning-top morphology composed of two parts, the upper part (episphere), which corresponds to the future prostomium, and the lower part (hyposphere) corresponding in part to the future pygidium. The morphology of the trochophore will considerably change during metamorphosis (Abeloos 1956). This developmental phase corresponds to the accumulation of mesoblast cells in the upper part of the hyposphere; these cells proliferate actively and push upward the new cells, which will organize in separate segments during the first steps of metamerization (Malaquin 1893; Herpin 1926; Allen 1964; Fischer et al. 2010). Then, this planktonic larva will settle on benthos (juvenile phase) where it begins to lengthen by the addition of segments (corresponding to metameres) to finally constitute a worm made of many segments between the pygidium and the prostomium. The proliferating zone of the young worm appears to function like the SAZ of the adult worm. Because of the continuous segment addition during the embryonic and adult life of most polychaete annelids, it is not surprising that these worms are able to regenerate posteriorly (PR for posterior regeneration) and use a similar mechanism as during embryonic development, and particularly a SAZ to regenerate the lost segments (Figs. 1B, 2A).

During development, the mesodermal cells of the larval posterior growth zone originate from the 4d micromere as well as embryonic segmental mesoderm coming from teloblastic divisions (Özpolat et al. 2017). However, during regeneration, the nature of the mesodermal cells in the blastema that give rise to the SAZ of adult annelids needs further research. Are they pluripotent cells, similar to those present during the embryonic development, or are they differentiated cells? Which of them is able to be stimulated to proliferate and how? Mesodermal blastema cells need now to be analysed at the level of cell lineage as it was done in embryos; their culture seems also necessary to be used in order to clarify the role of factors which control their proliferation in the SAZ, a method which was used in the past on another regeneration model (Boilly and Albert 1988). New techniques, such as single-cell transcriptomics, are currently providing interesting results on regeneration abilities and cell type diversity in the clitellate annelid *Pristina leidyi* (Álvarez-Campos et al. 2023). However, this species is not closely related to the model annelids investigated herein (Nereididae, Syllidae and Aricidae), and how well-conserved are cell types and molecular pathways across the phylum are still unclear.

## Segment addition zone (SAZ) and regeneration

The SAZ corresponds to a thin transversal region formed by two rings of proliferating cells, ectodermal and mesodermal, respectively (Balavoine 2014). After posterior segment amputation, worms first regenerate a pygidium immediately posterior to the last segment of the stump, and then a SAZ forms adjacent to the regenerated pygidium (Fig. 2A), which produces new segments at a fast initial rate and then slows down. An active SAZ always remains posterior to the most recently formed segment, and thus regenerated segments will show a postero-anterior differentiation gradient, with the most differentiated segment being always adjacent to the last segment of the stump (red arrow in Fig. 2A). The regeneration course follows a general common S-shape growth curve. For example, *Syllis amica* Quatrefages regenerated 10 segments during the 10 first days following the beginning of segmentation and around 13 segments during the next 40 days) (Boilly 1967a). After this period (namely around 60–70 days postamputation (dpa) for *S*. *amica*), regeneration is considered finished as the regenerated tail will continue to grow but at a slower pace corresponding to the normal growth of the worm (1–2 segments in 40 days). Therefore, in this species, posterior regeneration needs around 60–70 dpa to be completed (Boilly 1969a). This posterior regeneration, which mimics the posterior growth of the larva, operates similarly in all other annelids, although the regeneration time course may differ among species. Interestingly *S*. *amica*, as many other syllids, is characterized by the presence of a complex anterior digestive tube of stomodeal origin (Fig. 2B) and is able to regenerate anteriorly (AR) in opposition to many other annelids (Bely 2006; Hofmann 1975). But contrary to posterior regeneration (PR), *S*. *amica* does not regenerate the ectodermal foregut and endodermal midgut anteriorly, and the time course of segmentation is largely different from that of PR. Particularly, and although segmentation begins just after the differentiation of the prostomium, the production of new segments is relatively slow and stops at 45 dpa: at that time, the regenerate is composed of the prostomium and 3–4 segments (peristomium included) produced from a small proliferating zone adjacent to the first segment of the stump. Because of its position, this zone will be separated from the prostomium, which is pushed forward by the new segments, while in PR, the proliferating zone stays always adjacent to the pygidium (Fig. 2A). Nevertheless, the proliferating zone of AR functions as that of PR by producing new segments, and therefore can be considered as a transient SAZ in these species. The absence of regeneration of the intestine in AR after segment amputation, which represents the main difference between AR and PR, could explain the limited activity of this transient SAZ (Fig. 2E), as two other species of *Syllis*, *S*. *gracilis* (Boilly and Thibault 1974) and *S*. *malaquini* (Ribeiro et al. 2021), which have the particularity to regenerate anteriorly the stomodeal digestive tube (anterior digestive tube), regenerate more segments than *S*. *amica* (Fig. 2G and H). This is also the case in some rare other Syllidae as *Procerastea halleziana* (Langhammer 1928) and *Autolytus longeferiens* (now *Epigamia alexandri*) (Malaquin 1893).

Interestingly, the longitudinal discontinuity resulting from the juxtaposition of the regenerated pygidium to the last segment of the stump for PR, and of the regenerated prostomium to the first segment of the stump for AR, will be removed by segment addition resulting from the activity of each SAZ. This discontinuity is generally considered one of the initiators of regeneration (intercalary regeneration) and has been well studied during leg regeneration of amphibians and insects (review in Boilly et al. 1970). This “regeneration signal” (Abeloos 1932) has been interpreted in terms of positional information (Wolpert 1969), especially in amphibians (French et al. 1976) and also in planarians (Agata et al. 2007). Nevertheless, discontinuity is not enough to allow regeneration as the presence of regeneration cells and growth factors essential to sustain cell proliferation are necessary for segment regeneration. This has been experimentally shown in three models, *Syllis amica* Quatrefages, *Nereis diversicolor* O.F. Muller (currently *Hediste diversicolor*) and *Aricia foetida* Claparede (currently *Phylo phoetida*), which regenerate posteriorly (PR) in a similar way, although the speed of segmentation differs between these species (see below).

## Regeneration cells

In *Syllis amica,* all blastema cells originate from the amputated segment without intersegment migration, each cellular compartment of the blastema being produced from the corresponding tissue of the stump, a process involving partial dedifferentiation followed by redifferentiation (Boilly 1969a). In PR, the last segment of the stump is at the origin of blastema mesodermal cells (Fig. 1D); however, the posterior extremity of stump epidermis and intestinal epithelium supply blastema epidermal cells and intestinal epithelium blastema cells, respectively (Boilly 1967a). Nevertheless, we observed only one exception to this rule: when no intestinal epithelium is present, like in the stomodeum-derived anterior digestive tube (also called pharynx in syllids), it is regenerated from the stomodeal epithelium (of ectodermal origin) a process corresponding to a transdifferentiation (Boilly 1967b), a rare phenomenon which was also observed during the anterior gut regeneration of *S*. *malaquini* (Ribeiro et al. 2021) and Phoronida (Emig 1973).

Interestingly, several studies have shown that after X-ray irradiation of whole worms before or soon after amputation (Boilly 1969b), no segment regenerates. Selective complete destruction of mesodermal regeneration cells has no effect on regeneration of the pygidium but inhibits the SAZ. Similar results were obtained after coelomic microinjection of Thorotrast* (a suspension of radioactive thorium dioxide) into segments which were cut through several days after injection (Boilly 1969c). Cytological observations of treated worms confirmed that each of the two treatments affected coelomic epithelial cells, but not those of epidermal and intestinal epithelia. These results confirmed that the mesodermal regeneration cells are essential for the function of SAZ, while the regeneration of the pygidium and prostomium is independent of mesodermal regeneration cells. Additionally, incomplete destruction of mesodermal regeneration cells by X-irradiation or Thorotrast poisoning allows the regeneration of some segments whose number is inversely proportional to the X-irradiation dose or the Thorotrast concentration used (Boilly 1969b, 1969c).

Inhibition of SAZ function was also observed in worms deprived of the digestive tube after microdissection (or selectively poisoned with Thorotrast) (Boilly 1969d). However, it is not known if this effect results from the absence of the corresponding coelomic epithelium cells or from a hypothetic morphogenetic action of the digestive tube on PR; in *Nereis* sp., we observed that deviating a cut intestine into the dorsal side of the worm, without segment amputation, can induce the regeneration of a dorsal tail (Boilly 1973). This result could suggest a morphogenetic action of the digestive tube on regenerates; however, in this case, we created a contact between epidermal cells of the tegument and intestinal epithelial cells in the same manner as when we amputate segments, a situation which initiates posterior regeneration. Moreover, the influence of the digestive tube on the SAZ function could also be related to the presence of many nerve fibres in the gut wall’s nerve plexus.

Selective destruction of mesodermal regeneration cells in AR has no effect on the regeneration of the prostomium but inhibits segment regeneration (as in PR). Therefore, worms deprived of mesodermal regeneration cells are always able to regenerate the pygidium or the prostomium but are unable to regenerate segments, a result which indicates that the SAZ functions during PR or AR only if these regeneration cells are present. However, because it was not possible to deprive worms from their epidermal cells without severely altering their integrity and survival, we cannot exclude a role of the epidermal cells of the SAZ (Boilly 1967b, 1969a).

X-irradiation of amputated *Phylo foetida* (Boilly 1968b) and *Hediste diversicolor* (Boilly 1969e) gave the same results as in *Syllis amica*: the pygidium regenerated normally, but the regeneration of segments was inhibited. These results point out the importance of mesodermal regeneration cells in the function of SAZ, as well the high sensibility to X-irradiation of these cells, which might be in relation to their low differentiation linked to high proliferative activity. Some of these cells present a typical aspect of activated cells at the cytological level; they are characterized by a large nucleus and a big nucleolus, which is surrounded by a clear large area, and with a very basophil cytoplasm (Fig. 1D–F), corresponding to high rRNA synthesis (Boilly 1968a). Such activated cells were also described during the regeneration of clitellates (Bilello and Potswald 1974; Hill 1970; Bely 2014; Zattara and Bely 2013; Zattara et al. 2016) and called “neoblasts” (Randolph 1892). A term used as well for toti-pluripotent stem cells in planarians (Baguñà 2012; Karami et al. 2015; Reddien 2018), though these are substantially different to annelids neoblast-like cells (Boilly 1969f; Özpolat 2016).

## Nerve factors

Segment amputation leads to the rapid proliferation of nerve fibres posteriorly from the transected nerve cord toward the regenerated pygidium. These new nerve fibers creep between the epidermis and the mesodermal regeneration cells up to the regenerated pygidium where the mesodermal regeneration cells are well activated. However, during AR (in *Syllis* able to regenerate anteriorly), nerve fibres regenerate anteriorly toward the regenerated prostomium where the brain is differentiating; consequently, nerve cord fibres no longer regenerate close to the amputation plane, as is normally the case during PR. This is due to the differential position of each SAZ (close to the amputation area in AR, while close to the pygidium in PR, Fig. 2A). A similar observation was also reported in another syllid, *Typosyllis antoni* (Weidhase et al. 2017), a situation which could explain the rapid decrease of segment regeneration during AR. The role of the nerve cord in the function of SAZ cannot be demonstrated clearly as it is quasi-impossible to destroy it selectively. Nevertheless, it is possible to appreciate its role thanks to the observation of natural double nerve cords in the segment in some *Nereis* specimens (Boilly et al. 1975). Each nerve cord was in correspondence with a normal tail, giving sometimes 2 tails merging from the same segment. Moreover, the role of the nerve cord in the function of SAZ appears, in some cases (Nereidae), to be dependent of the endocrine action of the brain. Without the brain, pygidium regeneration is possible, but segmentation fails even in the presence of a normal nerve cord (Boilly 1974; Hofmann 1976; Alvarez-Campos et al. 2022). Nevertheless, this observation has to be interpreted carefully since it is well known that decerebration induces sexual maturation and, in most nereid species and many stolonizing syllids, a very important transformation of the worm corresponding to morphologic modifications in relation to spawning (Durchon 1952). It has been pointed out that these transformations monopolize energy to the detriment of regeneration and especially to segmentation (Golding 1974). An observation (Fig. 1C) of the regenerate of *Nereis* decerebrated before amputation showed nevertheless the first steps of segmentation (Boilly 1974). However, this segmentation could not be followed up since the worms died after spawning (Boilly-Marer 1976). Interestingly, in addition to the role of the nerve cord on regeneration and cell proliferation, and because of its function on signalling positional information (Pfannenstiel 1984; Boilly et al. 2022), the nerve cord also participates in the segmentation process. As the nerve cord is involved in AP polarity, the difference in AP polarity expression between AR *vs* PR could result from a limited action of the nerve cord during AR *vs* PR, a situation we already observed with the segmentation time-course during regeneration (Boilly 1969a). The use of AP polarity markers such as *Wnt*, or *Hox* genes, could provide a response to this question.

## Comparison between posterior and anterior regeneration

As the SAZ functions in PR in a similar way in *Hediste diversicolor, Phylo foetida* and *Syllis amica*, we will focus essentially on *Syllis amica* and other Syllidae species, which offer the opportunity to compare PR with AR. PR and AR include 2 phases: the first phase is the regeneration of the terminal part of the worm (the pygidium for PR, the prostomium for AR); the second phase is represented by the differentiation of segments from the SAZ, which corresponds to the metamerization of the regenerate. While the first phase needs around the same time to be achieved in PR and AR (9–10 dpa for *Syllis amica*), the timing of the second phase is different between PR and AR. In PR, it is characterized by its rapidity and relatively high number of regenerated segments, compared to AR (*e.g.* 23 segments are regenerated by 60/70 dpa in PR, while only 3–4 segments by 40/45 dpa in AR). Moreover, regenerative segment formation grades into normal growth segment formation at the end of PR, while segment formation stops in AR. The first phase (regeneration of the terminal part of the worm) differs from the second phase (metamerization of the regenerate), because it does not need the presence of mesodermal cells. In PR, the presence of epidermal and intestinal cells is sufficient to build a normal pygidium, a situation similar to what we observed in *Hediste diversicolor*, whose pygidium also regenerates after X-irradiation while segmentation is inhibited (Boilly 1969d), as well in *Phylo foetida* (Boilly 1968b). However, in AR of *S*. *amica* the epidermis alone is enough to regenerate the prostomium. The pygidium and the prostomium might represent the two parts of the trochophore larva before the formation of the mesodermal primordia. Thus, this first regeneration phase might reproduce what happens during the trochophore development (at least in those species with this kind of larva). These situations clearly show that the regeneration of the pygidium, as that of the prostomium, are independent from that of segments, and consequently is not linked to the activity of a SAZ during regeneration.

The second phase (metamerization of the regenerate) needs the presence of mesodermal regeneration cells. When these cells are selectively destroyed, either with an appropriate dose of X-rays or intracoelomic injection of Thorotrast, no segment is regenerated, while epidermal and intestinal epithelia are not affected. Mesodermal cells are vital for the segmentation of the regenerate. This condition is also found during the development of the trochophore, whose metamerization starts only after the appearance of mesodermal primordia. For lower X-ray dose, or diluted Thorotrast intracoelomic injection, segment regeneration was possible thanks to viable blastema mesodermal cells not killed by the treatment. This suggests that the number of regenerating segments depends on the prevalence of cell death after X-irradiation or Thorotrast injection (Boilly 1969b, 1969c). One of the first effects of mesodermal cell loss concerns the beginning of segmentation, which is delayed in proportion with the intensity of treatment both in AR and PR. However, the following segmentation course distinguishes PR from AR.

In PR, segmentation speed decreased with increasing X-ray dose. However, this loss of regenerated segments was not counterbalanced by a significative lengthening of the segmentation phase since regeneration stopped around 60–70 dpa in *Syllis amica* whatever the dose used (Boilly 1969b). This result shows that the extent of regeneration (number of regenerated segments) is not linked to the number of segments missing after amputation, as it was sometimes proposed (review in Abeloos 1932). The meaning of this regeneration stop is still not understood; the SAZ is always active as the normal growth takes over from regeneration but at a lower speed. The hypothetic “regeneration signal” (Abeloos 1932) resulting from the discontinuity between the regenerated pygidium and the last segment of the stump, with a lifespan limited to 60–70 days in our model, could initiate the regeneration process which should be maintained only during this time. Nevertheless, discontinuity is not enough to allow regeneration, as growth factors from nerves are necessary to sustain cell proliferation, as demonstrated in amphibians (Stocum 2017) and cancer models.

Although the beginning of segmentation was delayed in the same manner in AR as in PR, contrary to PR, segmentation showed to be less or not responsive to X-ray dose increase. Segmentation speed did not change significantly, and for all X-ray doses used (except for doses which stop segmentation both in PR and AR), the number of regenerated segments was the same (around 4). It is possible that the low capacity of AR is linked to a limited availability of mesodermal regeneration cells. Two reasons could explain this situation: (1) the absence of digestive tube participation in AR, (2) the limited stimulatory action of nerves on these cells. Indeed, we showed in *S*. *amica* that the digestive tube does not participate in AR, contrary to PR where the presence of the digestive tube is necessary for segmentation. We consider that the absence of a digestive tube in AR could lower the mesodermal regenerative cell population, as the coelomic epithelium surrounding the digestive tube is an important source of mesodermal regenerative cells. Some observations support this hypothesis. While *S*. *amica* is unable to regenerate the digestive tube anteriorly, two other syllids (*Syllis gracilis* and *Syllis malaquini*), which regenerate easily 14 anterior segments at 59 dpa (Boilly and Thibault 1974), and 8 segments 14 dpa, respectively (Ribeiro et al. 2021) can do it. They are even able to regenerate the whole anterior part of the digestive tube derived from the stomodeum (pharynx, proventricle, ventricle and its caeca). This situation was described also in 2 other Syllidae: *Procerastea halleziana* (Langhammer 1928) and *Epigamia alexandri*, which regenerate 10 segments anteriorly together with the complete anterior digestive tube (Malaquin 1893). These species show therefore stronger regenerative abilities, contrary to many other syllids like *S*. *amica*, which do not regenerate the digestive tube anteriorly and instead regenerate only a few segments (3–4 for *S*. *amica*). In PR, the situation is quite different because, contrary to AR, no segment is regenerated in the absence of the digestive tube (Boilly 1969d), a result which suggests that the morphogenetic function of this organ takes priority over its function as a source of mesodermal regeneration cells (Boilly 1973). This could also explain why PR decreased in relation to Thorotrast poisoning of intestinal cells (Boilly 1969d), a treatment which certainly reduced the regeneration of the digestive tube.

Nevertheless, if the presence of regeneration cells close to the wound is a prerequisite for regeneration, their multiplication is a necessity to the rebuilt of lost tissues after amputation. This might involve growth factors, and especially those released from nerves as described in other regeneration models (Sinigaglia and Averof 2019). Knowing the essential role of regenerating nerve fibres on proliferation during regeneration (Boilly and Bauduin 1988; Taban et al. 1996; Zenjari et al. 1997), as well as in cancer (Guo and Gil 2022), we consider that a difference of nerve activity in PR *vs* AR could explain the difference of function of the corresponding SAZ. In PR, regenerated nerve fibres from the amputated nerve cord are always in contact with mesodermal cells; consequently, mesodermal cells, always maintained in activity, constantly bring new cells for regeneration and normal growth. On the other hand, in AR, nerve fibres regenerate from the severed nerve cord into the blastema, as during PR, but rapidly connect with the newformed brain of the regenerated prostomium as it was already observed in another syllid (Weidhase et al. 2017). As a consequence, the number of regenerating nerve fibres decreases in AR-SAZ, which will be relatively denervated compared to the PR-SAZ. Therefore, without regenerating nerve fibres, the proliferation of mesodermal cells decreases correlatively with the activity of AR-SAZ, and regeneration ultimately stops. This would explain why AR ends largely before PR, the SAZ of which remains always functional, first for posterior regeneration, then for normal growth. Behind these observations, the nature of the nerve factor controlling the proliferation of regeneration cells (Sinigaglia and Averof 2019), as well as cancer cells (Guo and Gil 2022), needs to be known within the scope of regeneration and cancer control research.

These results mean that PR-SAZ functions differently from AR-SAZ. However, one could object that sexual maturation/stolonization, which is normally induced in posterior segments,—those used for AR study—could disturb AR (Durchon 1959, 1952). We checked this possibility on *S*. *amica* by using posterior segments re-amputated 50- and 75-days post-amputation, X-irradiated or not, and even after stolon release. All were able to regenerate anteriorly 3 to 4 segments at the same time, suggesting that sexual maturation did not disturb regeneration. Two factors (digestive tube, nerves) which play a role in the availability of mesodermal regeneration cells might explain the difference of SAZ function in PR vs AR. This problem warrants further investigations, especially at the molecular level. This was explored recently at the transcriptomics level on two syllid species: *Sphaerosyllis hystrix*, which exhibits limited AR and *Syllis gracilis* able to well regenerate anterior segments, and the entire part of the anterior digestive tube (Ribeiro et al. 2019). This study has shown that gene expression during PR and normal growth are similar but not identical to AR. However, this preliminary study does indicate the genes more particularly concerned with PR vs AR, and more investigations are needed to know which gene(s) are involved in the different regeneration phases, and how they can be modulated in experimental situations to clarify how they participate in regeneration.

## Conclusions and further directions

In polychaete annelids, segment amputation first prompts a rapid healing, and then regeneration of the pygidium (in PR) or the prostomium (in AR) on the amputation plane adjacent to the first proximal segment of the stump. This situation creates a positional discontinuity between the pygidium (or the prostomium) and this segment, discontinuity which is supposed to initiate the regeneration of the lost part of the worm by the production of new segments. This process requires the presence of a SAZ which will appear adjacent to the regenerated pygidium (for PR) or the first segment of the stump (for AR). Experimental results related to segment regeneration suggest that this SAZ functions differently in PR vs AR.

The SAZ function might depend on the availability of mesodermal regenerative cells, which are produced from the coelomic epithelia of the amputated segment. AR in *S*. *amica* might be characterized by the non-involvement of the digestive tube in regeneration (the consequence of which is a deficit in mesodermal cells) and a nerve activity limited in time (the consequence of which is a decrease of proliferation in mesodermal regeneration cells). On the other hand, PR might take advantage (1) of the participation of the digestive tube and particularly of its intestinal coelomic epithelial cells representing the main source of mesodermal regeneration cells, and (2) of the regenerated nerve fibres from the severed nerve cord and regenerated neurons, which stimulate the proliferation of the mesodermal regeneration cells during the whole regeneration process, and the following normal growth. These results highlight the importance of the regulation of mesodermal regeneration cell availability, though more evidence, requiring finer mechanistic and molecular testing is still necessary.

Regarding the regeneration of the nervous system, it is interesting to note that AR and PR show important differences. In AR, the outgrowths from the nerve cord form a loop that will ultimately differentiate into the circumenteric connectives and brain, hence becoming circular and “closed”, thus unable to release further growth factors. In contrast, in PR, the posterior end of the nerve cord remains next to the SAZ, and stays “open”, developing the VNC at the forming segments. This situation might allow a constant releasement of the putative growth factors. This hypothesis might be interesting to be further experimentally tested. For example, the nerve fibers of an anteriorly regenerating worm could be “kept open” by microsurgery or laser ablation to see if this results in enhanced regeneration of anterior segments.

## Data Availability

Not applicable

## Abbreviations

AR: Anterior regeneration
dpa: Day post amputation
PR: Posterior regeneration
SAZ: Segment addition zone
SEM: Scanning electron microscopy
TEM: Transmission electron microscopy
